# Transition rates to schizophrenia and early intervention effectiveness in substance-induced and brief psychotic disorders: a randomized controlled trial

**DOI:** 10.1038/s41598-025-24785-z

**Published:** 2025-11-18

**Authors:** Mohamed Abouzed, Amgad Gabr, Mohamed Elsheikh, Khaled A. Elag, Mohamed Y. Zeid, Mostafa Barakat, Ehab Elasaly, Nisrin Elsaadouni, Mahmoud Soliman

**Affiliations:** 1 Psychiatry Department, Faculty of Medicine, Al-Azhar University, Cairo, Egypt; 2 Psychiatry Department, Faculty of Medicine, Ain Shams University, Cairo, Egypt; 3 Psychiatry Department, Faculty of Medicine, Al-Azhar University, Assiut, Egypt; 4 Psychiatry Department, Faculty of Medicine, Mansoura University, Mansoura, Egypt

**Keywords:** Diseases, Medical research, Risk factors

## Abstract

This multisite, prospective randomized controlled trial examined transition rates to schizophrenia in individuals with recent-onset substance-induced psychotic disorder (SIPD) or brief psychotic disorder (BPD), and evaluated the efficacy of a specialized early intervention (SEI) designed to reduce this transition. We enrolled 1,000 participants (aged 16–40 years) across five Egyptian centers, randomly allocating them to SEI (*n* = 502) or treatment-as-usual (TAU; *n* = 498). TAU included standard antipsychotic treatment (primarily risperidone or haloperidol), monthly psychiatric follow-ups, and case management without structured psychosocial components. SEI combined monthly family psychoeducation, weekly cognitive-behavioral therapy, and low-dose risperidone (2 mg/day) with therapeutic drug monitoring. At 2-year follow-up, 26.3% of participants transitioned to schizophrenia. Transition rates were significantly higher in SIPD (29.1%) than in BPD (20.4%; HR = 1.48, *p* = 0.008), particularly among those with cannabis-associated SIPD (38.1%). SEI was associated with a 39% reduction in transition risk compared to TAU (HR = 0.61, *p* = 0.008). Key predictors included neurocognitive deficits (verbal learning OR = 0.79; working memory OR = 0.83), severe positive symptoms (OR = 1.15), and elevated inflammatory markers (CRP OR = 1.39). These findings suggest that SEI may reduce conversion, although causality cannot be definitively established. The study highlights the substantial risk of schizophrenia following a first psychotic episode, especially in the context of substance use. A comprehensive, multi-component early intervention appears effective in reducing transition rates, underscoring the importance of targeted preventive strategies.

## Introduction

 Psychotic disorders represent a major public health challenge, affecting approximately 3.89 to 4.03 per 1,000 individuals worldwide^1^. These conditions are characterized by a constellation of symptoms, including hallucinations, delusions, and disorganized thinking (positive symptoms), as well as social withdrawal, affective flattening, and avolition (negative symptoms)^2^. The personal and societal burden is substantial, with significant impacts on quality of life, functional capacity, and healthcare utilization^4^.

Defining first-episode psychosis (FEP) remains clinically challenging due to the often-insidious onset of symptoms and frequent delays in seeking treatment. Researchers have employed various operational definitions, including duration of untreated psychosis, first contact with mental health services, or initiation of antipsychotic medication^3^.

Outcomes after FEP are heterogeneous, with some individuals experiencing remission and others progressing to schizophrenia. Understanding predictors and interventions that may modify transition risk in acute or remitting psychoses is critical^5^. Meta-analytic evidence suggests that approximately 58% of patients achieve remission and 38% attain functional recovery after FEP^6^. However, relapse rates approach 80% within five years, and each recurrent episode may diminish the potential for full recovery^5^. Of particular concern is the subgroup of patients who transition to schizophrenia spectrum disorders, with systematic reviews estimating this progression occurs in 20–30% of cases within two years of initial presentation^7,8^.

Multiple risk factors for transition to schizophrenia have been identified through longitudinal research. Neurocognitive deficits—particularly in verbal learning, working memory, and executive functioning—represent robust predictors of diagnostic progression^9^. The severity of positive symptoms at initial presentation, as measured by instruments like the Positive and Negative Syndrome Scale (PANSS), shows consistent associations with transition risk^10^. Substance-induced psychotic disorder (SIPD), especially cannabis-associated, has drawn attention; however, substance use may act as a precipitating factor in genetically predisposed individuals rather than a direct cause^11^. Family history of psychotic disorders and certain inflammatory biomarkers, such as elevated C-reactive protein (CRP) and interleukin-6 (IL-6), have also been implicated in transition risk^12,13^.

Current treatment approaches demonstrate variable effectiveness in preventing diagnostic progression. While antipsychotic medications effectively reduce acute psychotic symptoms and prevent relapse in the short term, their long-term impact on transition rates remains uncertain^13^. Psychosocial interventions, including cognitive behavioral therapy and family psychoeducation, have shown efficacy in FEP but have not been widely studied in SIPD or brief psychotic disorder (BPD)^14,15^.

These limitations highlight critical gaps in our understanding of:

(1) the neurobiological mechanisms underlying transition from FEP to schizophrenia, (2) the optimal components and timing of early intervention strategies, and (3) the differential risk associated with specific substances of abuse.

The current study was designed to address these knowledge gaps by examining transition rates to schizophrenia in a large, well-characterized cohort of individuals with recent-onset SIPD or BPD. Using a comprehensive assessment protocol—including standardized clinical measures, neurocognitive testing, and inflammatory biomarkers—we aimed to: 1- Compare 2-year transition rates in SIPD and BPD. 2- Assess whether specialized early intervention (SEI) is associated with reduced transition risk compared to treatment as usual (TAU). 3- Identify predictors of transition independent of intervention effects.

Our findings provide important insights into the clinical course of early psychosis and inform the development of targeted preventive strategies for high-risk individuals.

## Methods

### Study design and participants

The *Psychosis Risk in Substance-Induced and Brief Psychosis (PRISM)* Study was a prospective, long-term, multi-center observational study with an embedded randomized controlled trial. It was conducted at five locations in Egypt: three Al-Azhar University hospitals (Cairo, Assiut, and New Damietta), and two additional hospitals in (Mansura city and Cairo city). A total of 1,008 patients aged 16 to 40 years were recruited.

Participants were eligible for inclusion if they had a DSM-5 diagnosis of substance-induced psychotic disorder (SIPD) or brief psychotic disorder (BPD) within the past three months and demonstrated the capacity to provide informed consent^16^. Exclusion criteria included a present or past diagnosis of schizophrenia or another enduring psychotic disorder, any neurological disorder or medical condition affecting brain function, and severe substance use disorder requiring inpatient detoxification. All participants underwent comprehensive baseline evaluations. Diagnostic confirmation was conducted using the Structured Clinical Interview for DSM-5 (SCID-5)^17^, and symptom severity was assessed via the Positive and Negative Syndrome Scale (PANSS)^18^. Neurocognitive functioning was evaluated using the MATRICS Consensus Cognitive Battery (MCCB), which measured verbal learning, working memory, processing speed, and social cognition, while premorbid IQ was estimated using the Wechsler Abbreviated Scale of Intelligence (WASI)^19,20^. Blood samples were collected to assess inflammatory biomarkers, specifically C-reactive protein (CRP) and interleukin-6 (IL-6), using enzyme-linked immunosorbent assay (ELISA) kits from R&D Systems (Minneapolis, MN). Substance use patterns were evaluated using the Timeline Follow-Back method (Sobell & Sobell,1992), supplemented by validated screening instruments for alcohol and drug use^21^. Following these assessments, participants were randomly assigned in a 1:1 ratio to either the Specialized Early Intervention (SEI) group or the Treatment-as-Usual (TAU) group using permuted block randomization stratified by site. The SEI group received a structured intervention that included monthly family psychoeducation sessions for one year, weekly cognitive behavioral therapy (CBT) sessions for six months targeting negative symptoms, social skills, and relapse prevention, and a fixed low-dose regimen of risperidone (2 mg/day) for one year, selected for its efficacy, tolerability, and affordability^22^. In contrast, the TAU group received standard care according to local protocols, which included antipsychotic medications (primarily risperidone, aripiprazole, olanzapine, or haloperidol at the clinician’s discretion), monthly psychiatric follow-ups, and case management, but did not include structured psychotherapy, family psychoeducation, or cognitive remediation. Participants completed standardized follow-up evaluations at 6-month, 1-year, and 2-year intervals. These assessments included PANSS ratings and SCID-5 interviews to monitor diagnostic transition to schizophrenia, condensed MCCB testing for neurocognitive performance, CRP and IL-6 biomarker analysis from blood samples, and substance use tracking using the Timeline Follow-Back method and validated screening tools.

To optimize retention, we implemented monthly appointment reminders, transportation assistance, and flexible scheduling. For participants unable to attend in person due to geographic or health constraints, telepsychiatry alternatives were offered via secure video conferencing. These remote sessions mirrored in-person protocols in content and duration. Caregiver involvement was encouraged across both formats to enhance treatment fidelity.

Although therapeutic drug monitoring via serum levels was unavailable, adherence was verified through monthly pill counts, structured self-reports, and caregiver confirmation when needed.

### Sample size and statistical analysis

The study was powered for a sample size of *N* = 1,000, with 500 participants allocated to each group, based on meta-analytic data indicating a 25% transition rate to schizophrenia within two years in the treatment-as-usual (TAU) group^23^. With 90% power to detect a 10% difference in transition rates between the SEI and TAU groups, the alpha level was set at 0.05 (two-sided). Descriptive statistics were used to summarize demographic and baseline characteristics. The primary analysis employed a random-effects mixed model to assess transition rates while accounting for site variability. To identify predictors of transition, logistic regression was conducted using baseline neurocognitive, biomarker, and clinical variables. Time-to-event data were analyzed using a Cox proportional hazards mixed-effects model, with study site included as a random effect. Group comparisons at baseline were performed using chi-square (χ²) tests and independent t-tests, and multivariable logistic regression was used to evaluate the influence of key predictors on transition outcomes.

### Ethical considerations

The research protocol was approved by the Al-Azhar University Institutional Review Board (IRB) Medical Center on January 17, 2021. All participants provided written informed consent prior to enrollment.

Participants were closely monitored for clinical deterioration and referred for more intensive treatment when necessary. The consent process included a thorough explanation of study procedures, risks, benefits, and the right to withdraw at any time. Data confidentiality and anonymity were emphasized. All procedures adhered to the Declaration of Helsinki. Copies of signed consent forms were provided to participants and retained by the research team.

## Results

### Participant characteristics

**Fig. 1:**
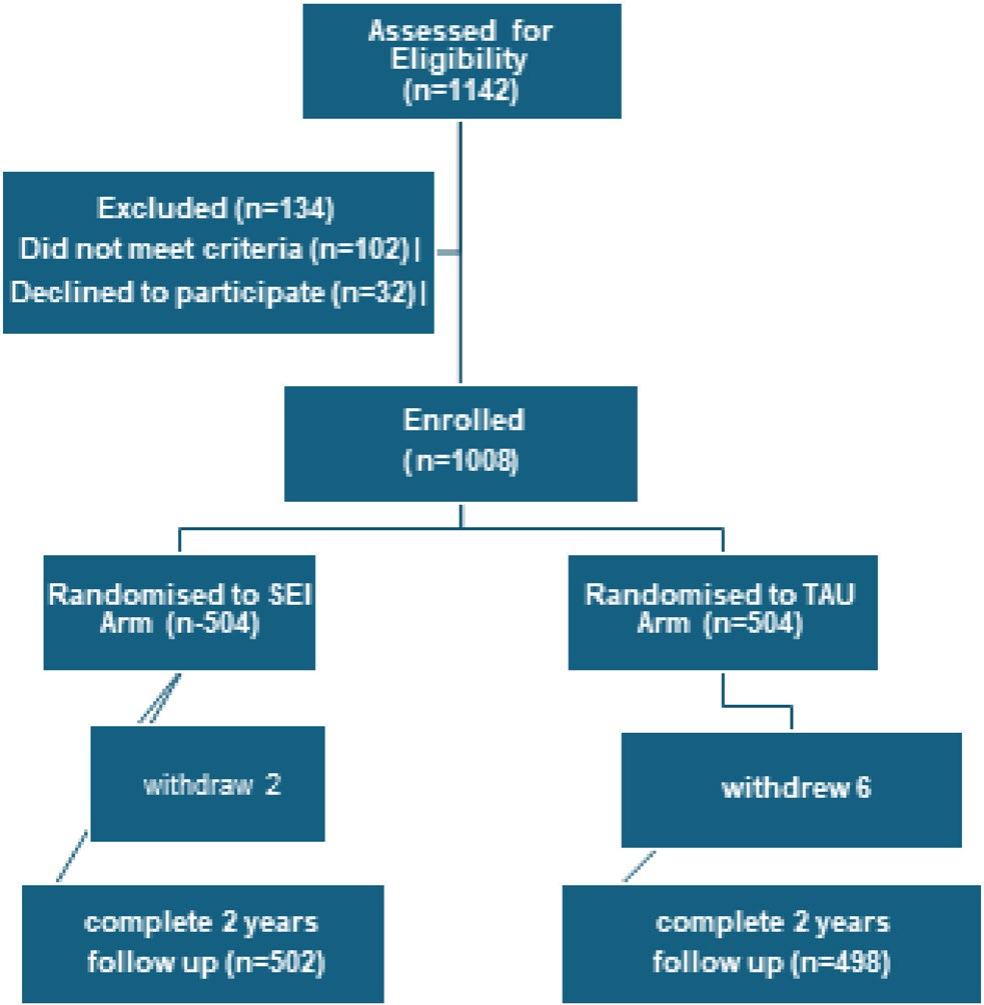
Flow chart.

A total of 1,142 individuals were screened for eligibility across the five participating sites. Of these, 1,008 met all inclusion criteria, resulting in an eligibility rate of 88.3%. Following baseline assessment, 8 participants withdrew, yielding a final sample size of *N* = 1,000. Stratified randomization ensured comparable demographic and clinical characteristics between the SEI and TAU groups. The average age was 25.2 years, with 61% of participants being male (Fig. 1).

### Baseline characteristics

The primary diagnosis was substance-induced psychotic disorder (SIPD) in 68% of participants (*n* = 677) and brief psychotic disorder (BPD) in 32% (*n* = 323). Cannabis was the most frequently implicated substance in SIPD cases (43%), followed by alcohol (22%) and amphetamines (19%). The median duration of the initial psychotic episode prior to baseline was 6 weeks (IQR = 3–12 weeks).

Comorbidities: 33% of participants (*n* = 330) had a co-occurring non-psychotic psychiatric diagnosis, with depressive disorders being the most prevalent (18%). 22% (*n* = 216) had a pre-existing substance use disorder prior to the onset of psychosis.

Participants with SIPD exhibited significantly higher transition rates to schizophrenia (29.1%) compared to those with BPD (20.4%; HR = 1.48, 95% CI: 1.11–1.98, *p* = 0.008). This difference was especially pronounced in cannabis-associated SIPD cases, which showed the highest transition risk (38.1% vs. BPD: 20.4%, *p* < 0.001). (See Table 1 for detailed baseline characteristics.)

**Table 1:** Participant characteristics.

Characteristic	Total (*N* = 1,000)	SEI (*n* = 502)	TAU (*n* = 498)	*p*-value
Age, mean (SD) years	25.2 (6.1)	25.0 (6.2)	25.4 (6.0)	0.32
Male sex, %	61%	60%	62%	0.54
SIPD, %	68%	69%	67%	0.48
- Cannabis-associated, %	43%	41%	45%	0.22
- Alcohol-associated, %	22%	24%	20%	0.12
- Amphetamine-associated, %	19%	20%	18%	0.41
BPD, %	32%	31%	33%	0.48
Substance use disorder, %	22% *comorbidities included*	20%	24%	0.11
Depressive disorders, %	18%	17%	19%	0.38
Overall transition rate, %	26.3%	20.1%	32.5%	< 0.001
- SIPD transition rate, %	29.1%	23.4%	35.8%	< 0.001
- BPD transition rate, %	20.4%	14.2%	26.6%	< 0.001

Transition to Schizophrenia: Over the 2-year follow-up period, the primary outcome—transition to schizophrenia—was diagnosed using the SCID-5. A total of 263 participants (26.3%) transitioned to schizophrenia by month 24.

Transition Rates by Site: A random-effects meta-analysis was conducted to assess site-specific transition rates, synthesized using the Freeman-Tukey double arcsine transformation. The overall incidence rate was 25.7% (95% CI: 21.4%–30.1%), with moderate heterogeneity (I² = 49%). (See Table 2 for site-specific data.)

**Table 2:** Predictors of transition to schizophrenia.

Predictor	Odds Ratio	(95% CI)	*P*-value
Verbal learning (MCCB)	0.79	(0.69–0.91)	0.001
Working memory (MCCB)	0.83	(0.73–0.94)	0.004
Positive symptoms (PANSS)	1.15	(1.08–1.22)	< 0.001
Substance use disorder	1.92	(1.43–2.59)	< 0.001
CRP	1.39	(1.12–1.73)	0.003
IL-6	1.22	(1.07–1.40)	0.004

Transition Rates by Substance Type: Cannabis use was associated with the highest transition rate to schizophrenia among individuals with recent-onset SIPD, followed by amphetamines and alcohol. Other substances were linked to lower transition rates compared to these three (Table 3).

**Table 3:** Transition rates by site.

SITE	Transition rate	(95% CI)
Azhar Cairo	0.27	(0.21, 0.33)
Azhar Assuit	0.24	(0.18, 0.30)
Azhar Demitta	0.23	(0.17, 0.29)
Mansoura city hospital	0.19	(0.14, 0.25)
Cairo city hospital	0.33	(0.27, 0.39)
Pooled Rate	0.26	(0.21, 0.30)
Heterogeneity: I2 = 49% |		

Predictors of Transition: Multivariable logistic regression identified several significant predictors of transition to schizophrenia (Table 4):

**Table 4:** Transition rates by types of substance:.

Substance	Transition rate	95% confidence interval
Cannabis	38.1%	30.2% − 46.3%
Alcohol	27.8%	21.4% −34.9%
amphetamines	33.5%	26.7% − 40.9%
Other substances	22.7%	16.8% − 29.8%

Neurocognitive Deficits: Each standard deviation decrease in verbal learning (California Verbal Learning Test) was associated with a 21% reduction in odds of transition (aOR = 0.79, 95% CI: 0.69–0.91, *p* = 0.001). Poorer working memory (WAIS-IV Digit Span) also predicted increased risk (aOR = 0.83 per SD decrease, 95% CI: 0.73–0.94, *p* = 0.004). Clinical Symptom Severity: Each 5-point increase on the PANSS positive symptom subscale was linked to a 15% increase in transition odds (aOR = 1.15, 95% CI: 1.08–1.22, *p* < 0.001).

Substance Use Disorders: Nearly doubled the risk of transition (aOR = 1.92, 95% CI: 1.43–2.59, *p* < 0.001). Cannabis use showed the strongest effect (aOR = 2.38 vs. non-users, 95% CI: 1.89–3.01).

Inflammatory Biomarkers: Each 1 mg/L increase in CRP was associated with 39% greater odds of transition (aOR = 1.39, 95% CI: 1.12–1.73, *p* = 0.003). IL-6 levels showed a similar but slightly attenuated effect (aOR = 1.22 per pg/mL increase, 95% CI: 1.07–1.40, *p* = 0.004).

The model demonstrated strong discrimination (AUC = 0.81, 95% CI: 0.78–0.84) and good calibration (Hosmer-Lemeshow χ² = 7.32, *p* = 0.50), explaining 32% of the variance in transition outcomes (Nagelkerke R² = 0.32). All analyses were adjusted for age, sex, study site, and treatment arm.

### Effect of specialized early intervention (SEI)

A Cox proportional hazards model was used to evaluate the impact of SEI on transition rates. The SEI group showed a 39% reduction in transition risk compared to the TAU group (HR = 0.61, 95% CI: 0.42–0.88, *p* = 0.008).

**Fig. 2:**
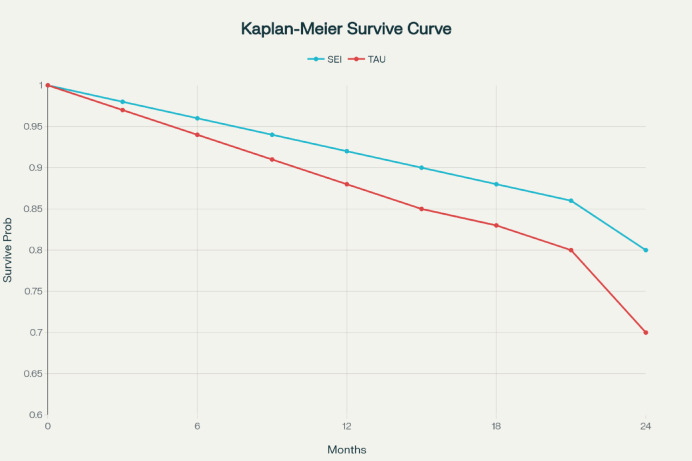
Kaplan-Meier curve of time to transition.

The Kaplan-Meier plot illustrates the cumulative probability of transitioning to schizophrenia over the 24-month period. The most substantial decline occurred within the first 12 months, with 75% of transitions occurring by month 16 (Fig. 2). The Kaplan-Meier plot illustrates the total likelihood of progressing to schizophrenia during the 24-month monitoring period. The most significant drop happens within the initial 12 months, with 75% of progress taking place by the 16th month following the baseline evaluation.

## Discussion

This multi-site prospective study provides valuable insights into transition rates to schizophrenia among individuals with recent-onset substance-induced psychotic disorder (SIPD) or brief psychotic disorder (BPD). Our findings demonstrate a pooled transition rate of 25.7% over the 24-month follow-up period, aligning with previous meta-analytic estimates reporting conversion rates between 20 and 30% within similar timeframes^8,23^. SIPD, particularly cannabis-associated, showed the highest transition risk. In line with current literature, we avoid causal inference and instead note that substance use may precipitate psychosis in predisposed individuals. The observed variation across studies—ranging from 11.3% in Swedish registry data to 46% in Finnish cohorts—likely reflects methodological differences in diagnostic criteria, sample characteristics, and follow-up duration^24,25^. Our use of stringent DSM-5 criteria via structured clinical interviews (SCID-5) and exclusion of cases with prolonged untreated psychosis may account for the intermediate transition rate observed.

Neurocognitive deficits, severe positive symptoms, comorbid substance use disorder, and elevated inflammatory markers predicted higher transition risk, independent of intervention group. The current results highlight neurocognitive deficits as significant predictors of diagnostic progression, with particular emphasis on impairments in verbal learning (OR = 0.79) and working memory (OR = 0.83), as measured by the MATRICS Consensus Cognitive Battery^20,26^. These findings extend previous research demonstrating the prognostic value of cognitive assessment in early psychosis^27^. It is important to note that while schizophrenia represents a serious mental health condition, contemporary outcome studies reveal substantial heterogeneity—with a meaningful proportion of individuals achieving functional recovery, particularly when comprehensive early intervention services are provided^6^.

The association between baseline positive symptom severity (PANSS scores) and transition risk (OR = 1.15) confirms established links between initial clinical presentation and long-term outcomes^10^. However, our results should be interpreted in light of emerging evidence that symptom severity represents only one dimension influencing recovery trajectories^28^. Our study also identified significant associations between elevated inflammatory markers (CRP OR = 1.39; IL-6 OR = 1.22) and progression to schizophrenia. These results align with growing evidence implicating inflammatory processes in the pathophysiology of psychosis^29,30^. The consistency of these findings across multiple studies suggests that peripheral inflammation may represent both a potential biomarker of transition risk and a modifiable therapeutic target in early psychosis interventions.

Specialized early intervention (SEI) was associated with a reduced risk of transition to schizophrenia over two years for both SIPD and BPD. The addition of family psychoeducation and CBT to standard pharmacological care provided plausible benefit, consistent with prior FEP research. The program demonstrated a 39% reduction in transition risk compared to treatment-as-usual (HR = 0.61), complementing findings from the Cochrane review of early intervention services^22^. Our comprehensive approach—combining low-dose risperidone (2 mg/day) with psychosocial interventions—showed benefits in service engagement and hospital admission rates, consistent with international standards for first-episode psychosis care^31^.

Regarding medication management, our use of fixed-dose risperidone was informed by both practical considerations (availability across Egyptian centers) and evidence supporting its efficacy in first-episode populations^22^. Contemporary network meta-analyses continue to support risperidone’s favorable efficacy-tolerability profile for first-episode psychosis^32^, particularly when considering: (1) D2 receptor occupancy studies showing 60–70% blockade at this dose—sufficient for therapeutic effect while minimizing extrapyramidal symptoms. (2) Metabolic risk profiles favoring risperidone over many widely available alternatives in our centers^33^.

### Limitations

Several limitations warrant consideration. First, our sample was drawn from Egyptian clinical centers, potentially limiting generalizability to other healthcare contexts. Second, the fixed-dose medication protocol, while methodologically necessary, differs from guideline-recommended approaches emphasizing individualized dosing. Third, substance use data relied partly on self-report, although supplemented with toxicology screening. Finally, the 2-year follow-up period precludes conclusions about longer-term outcomes.

### Future directions

Future research should aim to explore the underlying mechanisms through which Specialized Early Intervention (SEI) reduces the risk of transition to schizophrenia, shedding light on the specific therapeutic components that drive its effectiveness. Additionally, developing personalized interventions tailored to individual risk profiles—such as neurocognitive deficits, symptom severity, and patterns of substance use—may further enhance clinical outcomes. Longitudinal studies are also essential to evaluate the sustained impact of SEI on functional recovery and overall quality of life, providing a clearer picture of its long-term benefits in early psychosis care.

## Conclusion

This study underscores the substantial risk of transitioning to schizophrenia within two years following an initial psychotic episode. The findings highlight the importance of early identification and intervention in this population. Our results demonstrate the effectiveness of a standardized, multi-component early intervention in reducing the transition to schizophrenia, indicating the potential for targeted preventive strategies. The intervention—comprising pharmacological treatment, family psychoeducation, and cognitive behavioral therapy (CBT)—was associated with a reduced risk of transition for both SIPD and BPD. These findings suggest, though do not definitively prove, that comprehensive psychosocial strategies may improve prognosis. Further research is needed to optimize intervention components and to understand the long-term impact of early intervention on the course of psychosis.

## Data Availability

The data generated and analyzed during this study are available upon request. Please contact the corresponding author, Dr. Mohamed Abouzed, at dr_m.abozeid@azhar.edu.eg.
