# Correction: Transition rates to schizophrenia and early intervention effectiveness in substance-induced and brief psychotic disorders: a randomized controlled trial

**DOI:** 10.1038/s41598-026-37511-0

**Published:** 2026-01-29

**Authors:** Mohamed Abouzed, Amgad Gabr, Mohamed Elsheikh, Khaled A. Elag, Mohamed Y. Zeid, Mostafa Barakat, Ehab Elasaly, Nisrin Elsaadouni, Mahmoud Soliman

**Affiliations:** 1 Psychiatry Department, Faculty of Medicine, Al-Azhar University, Cairo, Egypt; 2 Psychiatry Department, Faculty of Medicine, Ain Shams University, Cairo, Egypt; 3 Psychiatry Department, Faculty of Medicine, Al-Azhar University, Assiut, Egypt; 4 Psychiatry Department, Faculty of Medicine, Mansoura University, Mansoura, Egypt

Correction to: *Scientific Reports* 10.1038/s41598-025-24785-z, published online 18 November 2025

The original version of this Article contained errors in the Methods sections under the subheading ‘Study design and participants’,

“The *Psychosis Risk in Substance-Induced and Brief Psychosis (PRISM)* Study was a prospective, long-term, multi-center observational study with an embedded randomized controlled trial. It was conducted at five locations in Egypt: three Al-Azhar University hospitals (Cairo, Assiut, and New Damietta), and two additional hospitals (Mansura University and Ain Shams University).”

now reads:

"The *Psychosis Risk in Substance-Induced and Brief Psychosis (PRISM)* Study was a prospective, long-term, multi- center observational study with an embedded randomized controlled trial. It was conducted at five locations in Egypt: three Al-Azhar University hospitals (Cairo, Assiut, and New Damietta), and two additional hospitals in (Mansura city and Cairo city).”

In addition, Table 3 contained errors, where the Site-Specific Transition Rates values were incorrect for “Mansoura” and “Ain Shams”. The correct and incorrect values appear below.

Incorrect:

**Table:** 

SITE	Transition rate	(95% CI)
Azhar Cairo	0.27	(0.21, 0.33)
Azhar Assuit	0.24	(0.18, 0.30)
Azhar Demitta	0.23	(0.17, 0.29)
Mansoura	0.19	(0.14, 0.25)
Ain Shams	0.33	(0.27, 0.39)
Pooled Rate	0.26	(0.21, 0.30)
Heterogeneity: I2 = 49% |		

Correct:

**Table:** 

SITE	Transition rate	(95% CI)
Azhar Cairo	0.27	(0.21, 0.33)
Azhar Assuit	0.24	(0.18, 0.30)
Azhar Demitta	0.23	(0.17, 0.29)
Mansoura city hospital	0.19	(0.14, 0.25)
Cairo city hospital	0.33	(0.27, 0.39)
Pooled Rate	0.26	(0.21, 0.30)
Heterogeneity: I2 = 49% |		

The original Article has been corrected.

